# Commentary: Blurring Borders: Innate Immunity with Adaptive Features

**DOI:** 10.3389/fmicb.2016.00358

**Published:** 2016-03-24

**Authors:** Edwin L. Cooper

**Affiliations:** Laboratory of Comparative Immunology, Department of Neurobiology, David Geffen School of Medicine, University of California, Los AngelesLos Angeles, CA, USA

**Keywords:** blurring, earthworm, innate, immune, memory

Adaptive immunity is now being deconstructed to encompass less stringent rules including initiation, and actual effector activity. Expanding the repertoire of invertebrate innate immunity has greatly facilitated a search for what actually constitutes ***innate*** and ***adaptive***. Strict definitions become **blurred** casting skepticism on using rigid definitions that define innate and adaptive immunity (Kvell et al., 2007). Immunology has experienced commendable growth. Immutable tenets deserve a brief mention. *First*, there is a need to limit strict divisions of ***adaptive* and *innate*** immunity. *Second*, to open these two views allows extended inclusions, reveals essential merits of innate immunity and admits inclusive invertebrate characteristics. We can even include features of adaptive responses especially to ***danger*** (Pradeu and Cooper, 2012). To facilitate this emerging reality recognizes hazy characteristics that fade into each other- that ***blur***; they are neither black nor white but a “clear gray”—reminiscent of impressionist paintings (Cooper, 2010, 2012).

***Blurring*** of immune responses has been confirmed as a distinct but related viewpoint. Removed from the pervasive T and B cell paradigm putative NK cells function in complex invertebrates (e.g., earthworms). For immunology during its youth, it was the NK cell that augmented intellectual understanding of immunity (Paust and von Andrian, 2011). Still perpetual bias prevailed, even ignoring immune response complexity in other vertebrates (fish, amphibians, reptiles, birds) and *per force* invertebrates! The *raison d'etre* for having evolved an immune response relegated functions akin to distinguishing ***self not self*** especially to external pathogens. But then the question arose questioning equally threatening internal threats, i.e., cancer. Predictably existence or lack of cancer may emerge as the last frontier as immunology perseveres.

Turning to a well-known invertebrate model, earthworm transplantation immunity is crucial. Xenografts from different genera are rejected suggesting responses that (1) mobilize immune clones; (2) react specifically against non-self antigens and retain memory (Cooper, 1969; Hostetter and Cooper, 1973). Clones may develop locally or be recruited and ***blurred***. Responses from danger signals may emanate from inflamed sites (Lemmi and Cooper, 1981; Pradeu and Cooper, 2012). Recipients of sensitized (immune, primed) coelomocytes reject test grafts more rapidly than controls, shorter than earthworms injected with leukocytes from unsensitized worms (Bailey et al., 1971). Memory is short lived, and occurs only if repeat grafts are transplanted before 10 days after the first immunizing grafts (Lemmi and Cooper, 1981; Cooper and Roch, 1986; Engelmann et al., 2011. Coelomocytes mediate rejection; tritiated thymidine (^3^HTdR) incorporates only into DNA of dividing cells. Coelomocytes divide or may be recruited after exposure to foreign antigen (Cooper and Roch, 1984). Recognition of, binding to, and killing foreign cells in a natural killer cell-like reaction reflects natural immunity (Cooper et al., 1995). Difference in ***responses*** between autogeneic and allogeneic effector cells may reflect interclonal ***immunologic rivalry*** causing ***blurring*** between incompatible effectors.

Earthworm coelomocytes (leukocytes) *in vitro* affect cytotoxicity against the NK-sensitive, human tumor cell line, K562, and the NK-resistant targets (U937, BSM, CEM). By cytofluorimetric analyses using mouse anti-human monoclonal antibodies, two coelomocyte types are: (1) small (8–11 micron) electron-dense cells (SC): CD11a+, CD45RA+, CD45RO+, CDw49b+, CD54+, beta 2-m+ and Thy-1+; (2) large (12–15 micron) electron-lucent cells (LC); they are negative for these markers, and for other CD and MHC class I and class II markers. SC are active during recognition, rapidly binding to targets; LC are phagocytic. Release of 51Cr revealed rapid, significant, and equal levels of killing; primitive NK-like activity evolved early (Cossarizza et al., 1996). This represents the first definition of distinction between cell killing and phagocytosis, so often misunderstood in invertebrate systems. Although T cells and B cells are absent in earthworms there is substantial evidence indicative of NK-like cells.

Natural killer (NK) cells are effector lymphocytes of innate immunity endowed with cytolytic functions. NK cells express a repertoire of activating and inhibitory receptors calibrated to ensure self-tolerance while allowing assaults against viral infection and tumor development. However, NK cells show no invariant response but rather adapt to their environment. Analyses unveil that NK cells mount a form of antigen-specific immunologic memory (Kurtz, 2005; Little et al., 2005). NK cells thus exert sophisticated biological functions attributable to innate and adaptive immunity, ***blurring*** any functional borders between these two arms of the immune response (Vivier et al., 2011). Attention is focused on lymphocytes that ***blur*** traditional boundaries between innate and adaptive immune systems (Figure 1, Criscitiello and de Figueiredo, 2013). The development and functional properties of “innate-like” B and T cells and natural killer (NK) cells augment understanding of innate lymphoid cells (ILCs; Lanier, 2013).

**Figure 1:**
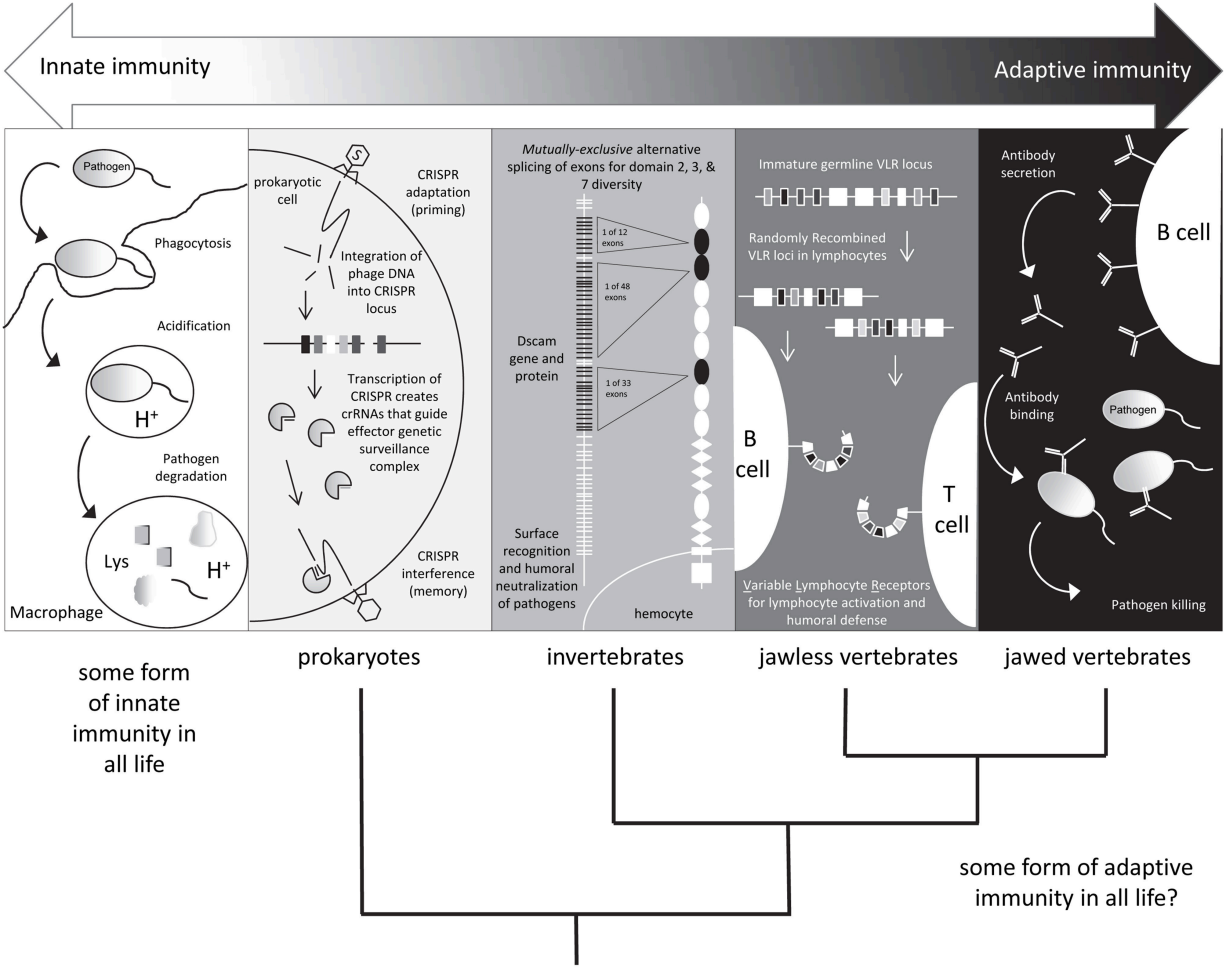
**Shades of immunity**. All life has innate immune mechanisms and jawed vertebrates have the IgSF lymphocyte receptor–based adaptive system as well. Different immune mechanisms with adaptive properties are being discovered in species originally considered to only possess innate immunity. Figure from Criscitiello and de Figueiredo (2013).

Invertebrate immunology and plant immunity have benefitted by understanding priming; it may actually be a template for designing future vaccines (Netea, 2013). No longer acceptable that innate is primitive or irreversibly non-specific, we can now achieve a prolonged, enhanced functional state after adequate **priming** by producing a new strategy: *Trained (innate) Immunity (TI*). By repetitive exposure or **priming**, TI can be important in host defense creating vaccine responses to certain diseases (van der Meer et al., 2015). TI results from epigenetic reprogramming of innate immune cells and ensures protective, non-specific effects previously induced by vaccines e.g., BCG, measles and whole-microorganisms (Blok et al., 2015). The inability of innate immunity to reliably build on memory is a main difference with the more durable adaptive immunity. Thus, a lasting state of significantly enhanced innate immunity, i.e., *trained immunity* can be mediated by prototypical innate immune cells i.e., NK cells and monocytes/macrophages, descendants of ancient phagocytes. Immediately practical, phytopathogens threaten food supplies and global food security; invertebrates are popular sources of food for humans and aquatic organisms in the food chain. Understanding pathogenesis and effector biology translates into new facilitating tools essential for developing durable disease resistance (Nejat et al., 2016). This represents an encouraging paradigm change in our concept of immunity and a more bountiful *landscape* spawned by priming and blurred responses!

## Author contributions

The author confirms being the sole contributor of this work and approved it for publication.

### Conflict of interest statement

The author declares that the research was conducted in the absence of any commercial or financial relationships that could be construed as a potential conflict of interest.
